# Movement correction in DCE-MRI through windowed and reconstruction dynamic mode decomposition

**DOI:** 10.1007/s00138-017-0835-5

**Published:** 2017-04-06

**Authors:** Santosh Tirunagari, Norman Poh, Kevin Wells, Miroslaw Bober, Isky Gorden, David Windridge

**Affiliations:** 1 Department of Computer Science, University of Surrey, Guildford, Surrey GU2 7XH UK; 2 Center for Vision, Speech and Signal Processing (CVSSP), University of Surrey, Guildford, Surrey GU2 7XH UK; 3 University College London (UCL) Institute of Child Health, 30 Guildford Street, London, WCIN 1EH UK; 4 Department of Computer Science, Middlesex University, The Burroughs, Hendon, London, NW4 4BT UK

**Keywords:** DMD, W-DMD, R-DMD, WR-DMD, DCE-MRI, Movement correction

## Abstract

Images of the kidneys using dynamic contrast-enhanced magnetic resonance renography (DCE-MRR) contains unwanted complex organ motion due to respiration. This gives rise to motion artefacts that hinder the clinical assessment of kidney function. However, due to the rapid change in contrast agent within the DCE-MR image sequence, commonly used intensity-based image registration techniques are likely to fail. While semi-automated approaches involving human experts are a possible alternative, they pose significant drawbacks including inter-observer variability, and the bottleneck introduced through manual inspection of the multiplicity of images produced during a DCE-MRR study. To address this issue, we present a novel automated, registration-free movement correction approach based on windowed and reconstruction variants of dynamic mode decomposition (WR-DMD). Our proposed method is validated on ten different healthy volunteers’ kidney DCE-MRI data sets. The results, using block-matching-block evaluation on the image sequence produced by WR-DMD, show the elimination of $99\%$ of mean motion magnitude when compared to the original data sets, thereby demonstrating the viability of automatic movement correction using WR-DMD.

**Electronic supplementary material:**

The online version of this article (doi:10.1007/s00138-017-0835-5) contains supplementary material, which is available to authorized users.

## Introduction

**Fig. 1:**
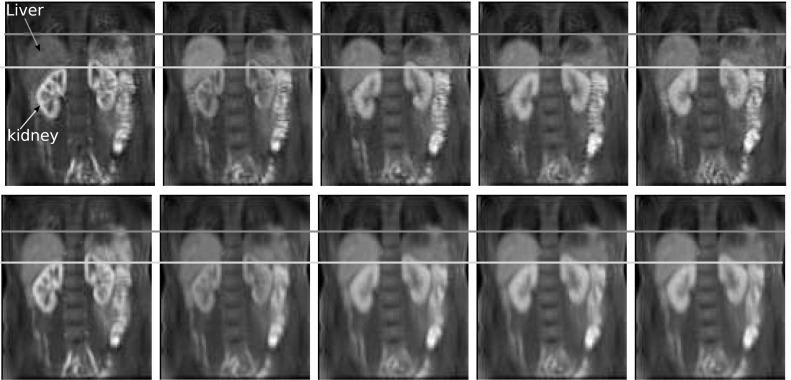
(*Top*) 5 frames at time = {30,50,74,82,100}s selected from a DCE-MRI sequence of a healthy volunteer. The *yellow* and *red reference lines* show the alignment of the kidney and liver regions. The *first image* shows the peak stage of contrast agent inside the kidney region. The regions of kidney and liver clearly depict the translation of movements in the vertical direction, arising due to the patient movements. (*Bottom*) The *yellow* and *red reference lines* clearly showing the proper alignment of the kidney and liver regions after processing with WR-DMD. The video-based results can be viewed at https://youtu.be/TWq34TFGNcU and https://youtu.be/UT7f4ch4H-I (colour figure online)

Dynamic contrast-enhanced magnetic resonance imaging (DCE-MRI) renography is a promising technique for functional assessment of the kidney because it avoids the need for ionising radiation. In order to obtain useful diagnostic/prognostic information, the dynamic change of pixel intensities as contrast agent flows through the metabolic region within the kidney must be quantified in terms of function such as blood flow, filtration rate or functional volume. However, absolute quantification inside the kidney is often obfuscated by complex patient movements, arising due to respiration, pulsation and involuntary movements as shown in Fig. 1 (top) (the corresponding dynamic sequence can be viewed at https://youtu.be/TWq34TFGNcU). These movements induce pixel displacements in and around the kidney region, leading to motion artefacts (intensity fluctuations) in the time–intensity curves produced from a fixed region of interest (ROI) placed within the kidney. Such motion artefacts can affect the assessment of the kidney function.

In order to correct for such motion, many rigid and non-rigid image registration techniques have been proposed in the literature [13, 37] for providing well-aligned features across the image sequence. However, developing a registration technique specific to DCE-MRI data is challenging due to the rapid change of contrast agent within the DCE-MRI sequence. Traditional registration techniques are likely to fail with DCE-MRI data as similar voxels in the sequence may have variable local intensities at each sampling time. Therefore, the algorithm has to be independent of the contrast changes within the kidney.

Senneville et al.’s [29] work assumes that the kidney is a rigid body, and its shape does not change during the MRI data acquisition. For registration purposes in Senneville’s work, a human expert selects a reference image from the image sequence. The expert then manually delineates the kidney region of interest (ROI), thus forming the ‘template’. Using this template, the registration of the kidneys is conducted across the DCE-MRI sequence using contrast invariant similarity matching. Other methods that require human input are reported in  [6, 15, 21, 36]. Although these approaches are potentially effective, a major issue is the need for human intervention for delineating the kidney region. Other issues are reproducibility and the intrinsic bottleneck associated with the speed of processing that automation could address.

On the other hand, approaches based on matrix decomposition such as principle component analysis (PCA), independent component analysis (ICA) and robust PCA (RPCA) have also been proposed as a preprocessing step prior to image registration. Progressive principal component registration (PPCR) introduced by Melbourne et al. [17, 18] is a PCA-based approach that iteratively removes misalignment from the DCE-MRI sequence while using a standard registration algorithm such as fluid registration [5]. The author’s main assumption in this work is that PCA captures contrast changes or intensity fluctuations in the first few principal components and motion in the last principal components (when sorted according to their proportion of variance explained through the cumulative sum of eigenvalues). However, using PCA for motion compensation depends purely upon the nature of the motion, i.e. for example, periodic motion of free breathing can appear in the first few principal components along with contrast changes.

In order to deal robustly with various breathing protocols, robust data decomposition registration (RDDR) [9] was introduced. RDDR uses robust principal component analysis (RPCA) [4] coupled with a registration algorithm based on residual complexity minimisation [20]. RPCA decomposes the DCE-MRI data into a series of low-rank and sparse components separating motion components from the contrast enhanced. The intensity fluctuations which remain unchanged are then registered. The explicit separation of sparse components provide RPCA a greater degree of robustness when compared to a regular PCA-based approach. The ICA-based approach has also been used to decompose DCE-MRI data prior to registration in free breathing cardiac MRI [35]. In all of the aforementioned approaches, the main objective is to remove motion elements from the DCE-MRI time series while utilising image registration methods.

Spatio-temporal ICA (STICA) [10, 30] is one method that does not use any kind of registration procedure in its approach. According to STICA’s assumptions, free breathing, which induces the movement artefacts, is regarded as one of the independent processes, i.e. different regions in DCE-MRI that respond differently with respect to the contrast agent are assumed to be spatially independent, and are assumed to be temporally independent of each other [10]. Quantitative assessment in [10] using ROI analysis shows virtually no movement in either the first independent component or the second. The third independent component shows the movement artefacts. Limitations of this approach can include finding an optimal filter that can maximise the statistical independence of the these DCE signals over space and time simultaneously.

**Table 1:** Comparison of proposed methods with other movement correction approaches that are based on matrix decomposition methods

References	Method	Matrix Factorisation	Registration	Assumptions	Demerits/merits
Melbourne et al. [17, 18]	Progressive principal component registration (PPCR)	PCA	Multi-resolution FFD [19]	Contrast changes captured in the first few principal components and motion in the last principal components	Limitations in handling periodic motion of free breathing. Computationally expensive for using registration algorithm
Hamy et al. [9]	Robust data decomposition registration (RDDR)	RPCA	Residual complexity minimisation [20]	Sparse components separate motion components from the contrast-enhanced images	Computationally expensive. Nevertheless robust to various breathing protocols
Kiani et al. [10]	Online—STICA	ICA	None	Different regions in DCE-MRI respond differently, over the time are assumed to be independent	Computationally expensive for optimising filters that can maximise the statistical independence
Proposed method	WR-DMD	DMD	None	Sparse components separate motion components from the contrast-enhanced images. Contrast changes captured in the most significant dynamic modes and motion in the least significant	W-DMD disentangles periodic free breathing. DMD separates motion components in least significant modes. R-DMD reconstructs perfectly aligned sequence with the most significant modes. Computationally inexpensive

### Motivation: dynamic mode decomposition (DMD)

DMD was originally introduced in the area of computational fluid dynamics (CFD) [28], specifically for analysing the sequential image data generated by nonlinear complex fluid flows [25–27, 34]. The DMD decomposes a given image sequence into several images, called dynamic modes. These modes essentially capture different large-scale to small-scale structures (sparse components) including a background structure (low-rank model) [7]. DMD has gained significant applications in various fields [2, 3, 16], including for detecting spoof samples from facial authentication video data sets [33] and for detecting spoofed finger-vein images [31]. The advantage of this method is its ability to identify regions of dominant motion in an image sequence in a completely data-driven manner without relying on any prior assumptions about the patterns of behaviour within the data. Therefore, it is thus potentially well-suited to analyse a wide variation of blood flow and filtration patterns seen in renography pathology.

### Comparison with decomposition-based methods

The assumptions of our approach are borrowed from PCCR and RDDR methods. Similar to STICA our proposed WR-DMD technique is also an image registration-free approach. Comparisons with the aforementioned decomposition-based methods are made in Table 1.

### Our approach

**Fig. 2:**
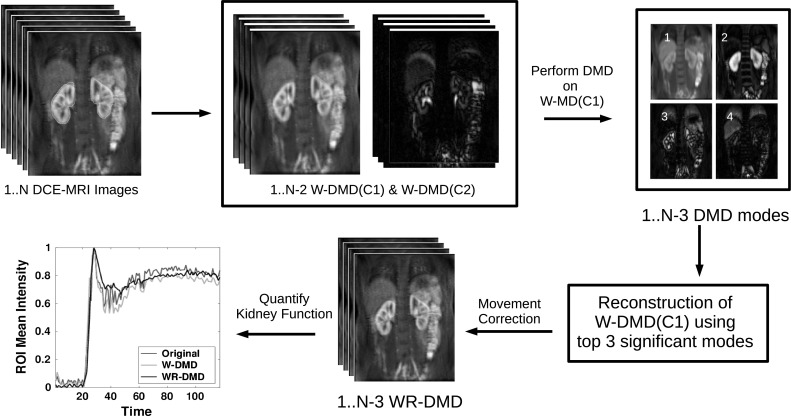
*Flow chart* showing the steps involved in the methodological framework. First, a DCE-MRI sequence consisting of *N* images is processed using the W-DMD algorithm in order to output each $N-2$ W-DMD components C1 and C2. At this stage, the W-DMD(C1) produces the low-rank images and W-DMD(C2) produces sparse images. W-DMD(C1) is given as an input to DMD which produces $N-3$ DMD modes. The first three DMD modes are then selected for reconstructing the motion-stabilised image sequence

**Fig. 3:**
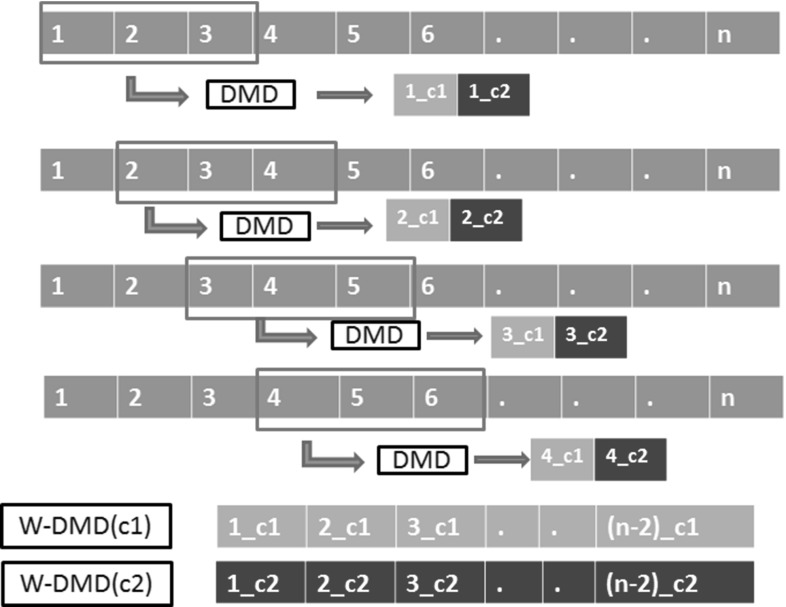
Methodological pipeline showing the working mechanism of W-DMD. DMD runs over the window containing first three images in the sequence, obtaining two dynamic modes. The first dynamic mode ‘c1’ capturing the low-rank image across the window and second dynamic mode c2 capturing the sparse representation. The next step exclude the first image and consider images $\{2, 3, 4\}$, followed by $\{3, 4, 5\}$ and $\{4, 5, 6\}$ producing c1 and c2 components. Finally, all of the c1s and c2s across all the windows are concatenated to obtain W-DMD component-1 (W-DMD (C1)) and W-DMD component-2 (W-DMD (C2))

Our approach is a two-step process (Fig. 2), where at first the DCE-MRI sequence is processed through window-DMD method [31] to compensate for the pseudo-periodic breathing motion (the importance of running W-DMD as a first step process is shown in Sect. 4.4). The windowed version of DMD method (here, $W\,=\,3$) runs over three consecutive images (since motion is periodic for every three images as evaluated in Sect. 4.2) in an overlapping fashion as shown in Fig. 3.

The output of DMD at each window produces two images namely W-DMD component-1 (C1) revealing the low-rank image and W-DMD component-2 (C2) revealing the sparse component. At this stage of our approach, we discard the sparse components, i.e. W-DMD(C2)’s to compensate the pseudo-periodic free breathing motion from the DCE-MRI sequence. Second, we proceed with giving W-DMD component-1’s as an input to standard DMD algorithm, which decomposes the W-DMD(C1) sequence into several images called dynamic modes (see, Fig. 8). The dynamic contrast changes are captured in the most significant modes, and motion components are captured in the least significant modes. Using the first three significant DMD modes, the original sequence is then reconstructed via R-DMD method. Our result in Fig. 1 (bottom) shows that the motion artefacts are compensated in an exemplar 4D dynamic medical imaging application.

### Contributions

Our implementation is novel in the sense that it uses windowed-DMD on dynamic image sequences for the first time to compensate for motion artefacts by producing low-rank images as W-DMD component-1. Even though the low-rank and sparse representations of an image sequence have been reported for DMD [7, 12], the method that we propose here is essentially different. In [7], the authors exploit the low-rank and sparse representation within each frame. Specifically, low rank revealing the background and sparse presenting the foreground of that particular frame. Recently, in [12] DMD with a multi-resolution approach (MR-DMD) decomposed video streams into multi-time scale features and objects. The MR-DMD approach is similar to that of applying standard DMD technique at several resolutions after discarding the slow varying modes (background modes or the most significant modes). In other words, the MR-DMD approach runs the standard DMD algorithm over a sequence of images to produce several dynamic modes. Later, the most significant modes (background modes) are discarded; therefore, DMD is run using the least significant modes. This process is continued at several time resolutions allowing an image sequence to be separated for objects moving at different rates against the slowly varying background. This approach, therefore, allows for multiple target tracking and detection. In contrast, the proposed W-DMD method runs standard DMD over a window of consecutive images; thus producing low-rank and sparse modes at each window of the image sequence, respectively, giving rise to W-DMD component-1 and component-2. The original DMD method introduced in [28] extracts modes from a sequence of images and interprets modes in the image space, whereas our reconstruction variant of the method (R-DMD) re-projects the DMD modes back into original image sequence, thereby stabilising the complex movements. Therefore, our contributions in this study are in (i) introducing the WR-DMD framework for the first time to reconstruct movement corrected, aligned images sequence. (ii) Validating our technique using medical data with applications to DCE-MRI; and (iii) improving the understanding of the application through WR-DMD framework.

### Organisation of the paper

The remainder of this paper is organised as follows: in Sect. 2, we consider the theory for WR-DMD. Sect. 3 presents 10 different data sets used in this study. In Sect. 4, we present our experiments and results, and finally, conclusions are drawn in Sect. 5.

## Methodology

In this section, we present our methodological pipeline which consists of W-DMD and reconstruction-based DMD. The overall process framework is shown in Fig. 2 and discussed in Sect. 1.3.

### Dynamic mode decomposition (DMD)

Let $\bar{\mathbf {x}}_r$ be the $r\mathrm{th}$ dynamic image frame in a DCE-MRI sequence, whose size is $m\times n$. This image frame $\bar{\mathbf {x}}_r$ is converted to $mn\times 1$ column vector, resulting in the construction of a data matrix $\mathbf X$ of size $mn \times N$ for *N* image frames.

1 $$\begin{aligned} \mathbf X = [\bar{\mathbf {x}}_1, \bar{\mathbf {x}}_2, \bar{\mathbf {x}}_3, \ldots , \bar{\mathbf {x}}_N] = \begin{pmatrix} x^{1}_1 &{} x^{1}_2 &{} \ldots &{} x^{1}_N \\ \vdots &{} \vdots &{} \vdots &{} \vdots \\ x^{mn}_1 &{} x^{mn}_2 &{} \ldots &{} x^{mn}_N \\ \end{pmatrix}. \end{aligned}$$

The images in the DCE-MRI data are collected over regularly spaced time intervals, and hence, each pair of consecutive images are correlated. It can be justified that a mapping *A* exists between them forming a span of krylov subspace [11, 22, 23]:

2 $$\begin{aligned}&\mathbf X = \left[ \bar{\mathbf {x}}_1, A\bar{\mathbf {x}}_1, A^2\bar{\mathbf {x}}_1, A^3\bar{\mathbf {x}}_1, \ldots , A^{N-1}\bar{\mathbf {x}}_{1}\right] .\nonumber \\&[\bar{\mathbf {x}}_2, \bar{\mathbf {x}}_3, \ldots , \bar{\mathbf {x}}_N] = A\left[ \bar{\mathbf {x}}_1, \bar{\mathbf {x}}_2, \ldots , \bar{\mathbf {x}}_{N-1}\right] .\nonumber \\&\mathbf {P}_2 = A\mathbf {P}_1 + \mathbf {re}^{T}_{N-1}. \end{aligned}$$

Here, ${\mathbf {r}}$ is the vector of residuals that accounts for behaviours that cannot be described completely by ${\mathbf {A}}$, $\mathbf {e}_{N-1} = \{0, 0 , 1\} \in \mathcal {R}^{N-1}$, $\mathbf {P}_2 = [\bar{\mathbf {x}}_2, \bar{\mathbf {x}}_3, \ldots , \bar{\mathbf {x}}_N]$ and $\mathbf {P}_1 = [\bar{\mathbf {x}}_1, \bar{\mathbf {x}}_2, \ldots , \bar{\mathbf {x}}_{N-1}]$. The system $\mathbf {A}$ is unknown and it captures the overall dynamics within the dynamic image sequence in terms of the eigenvalues and eigenvectors of $\mathbf {A}$, which are referred to as the DMD eigenvalues and DMD modes, respectively.

The sizes of the matrices $P_2$ and $P_1$ are both $mn \times N-1$ each. Therefore, the size of unknown matrix *A* would be $mn \times mn$. Since matrix *A* captures the dynamical information within the image sequence, solving it provides us with the dynamics captured in the image sequence in terms of modes. Unfortunately, solving for A is computationally very expensive due to it size. For instance, if an image has a size of $240 \times 320$, i.e. $m=240$ and $n=320$, the size of *A* is then $76800 \times 76800$.

From the literature, there are two approaches for obtaining these eigenvalues and modes. The first is Arnoldi-based approach, which is useful for theoretical analysis due to its connection with Krylov subspace methods [11, 22, 23]. The second is a singular value decomposition (SVD)-based approach that is more robust to noise in the data and to numerical errors [25].

Therefore, SVD can be directly applied on $\mathbf {\mathbf {P}_1}$ in Eq. (2), to obtain $\mathbf {U}$, $\varvec{\Sigma }$ and $\mathbf {V^*}$ matrices.

3 $$\begin{aligned} \mathbf {\mathbf {P}_2} = \mathbf {A U} {\varvec{\Sigma }} \mathbf {V^*}. \end{aligned}$$

$$\because \mathbf {\mathbf {P}_1}=\sum \limits _{i=1}^d{\varSigma }_i\mathbf {u}_i\mathbf {v}_i^{*}$$,

here, ${\varSigma }_{i}$ is the $i\mathrm{th}$ singular value of $\mathbf {P}_1$, $\mathbf {u}_{i}$ and $\mathbf {v}_{i}^*$ are the corresponding left and right singular vectors, respectively; and *d* is the total number of singular values.

Re-arranging Eq. (3), we obtain the full-rank matrix $\mathbf {{A}}$,

4 $$\begin{aligned} \mathbf {A} = \mathbf {P}_{\mathbf{2V}}{\varvec{\Sigma }}^{-\mathbf{1}}\mathbf {U^*}. \end{aligned}$$

Since the eigenvalue analysis is agnostic to any linear projection, solving the eigen problem of $\widetilde{\mathbf{H}}$ is easier than that of solving for $\mathbf {A}$ directly. Moreover, the associated eigenvectors of $\widetilde{\mathbf{H}}$ provide the coefficients for the linear combination that is necessary to express the dynamics within the time-series basis.

5 $$\begin{aligned} \widetilde{\mathbf{H}}\omega = \sigma \omega , \end{aligned}$$

where $\omega$ are the eigenvectors and $\sigma$ a diagonal matrix containing the corresponding eigenvalues of $\widetilde{\mathbf{H}}$ matrix. The eigenvalues of $\widetilde{\mathbf{H}}$ approximate some of the eigenvalues of the full system $\mathbf {A}$ [8], and we then have:

6 $$\begin{aligned} \begin{aligned}&\mathbf {AU} \approx \mathbf {U}\widetilde{\mathbf{H}},\\&\mathbf {AU} \approx \mathbf {U} \omega \sigma \omega ^{-\mathbf{1}},\\&\mathbf {A}(\mathbf{U} \omega ) \approx \mathbf {(U} \omega \mathbf{)}\sigma .\\ \end{aligned} \end{aligned}$$

Therefore, $\widetilde{\mathbf{H}}$ is determined on the subspace spanned by the orthogonal singular basis vectors $\mathbf {U}$ obtained via $\mathbf {\mathbf {P}_1}$,

7 $$\begin{aligned} \begin{aligned} \widetilde{\mathbf{H}}&= {\mathbf {U^{*}(A)U}},\\ \widetilde{\mathbf{H}}&= \mathbf {U^{*}}\left( \mathbf {P}_{\mathbf{2V}}{\varvec{\Sigma }}^{-\mathbf{1}}\mathbf {U^{*}}\right) \mathbf{U}, \end{aligned} \end{aligned}$$

which can be rewritten as:

8 $$\begin{aligned} \widetilde{\mathbf{H}} = \mathbf {U^{*}}\mathbf {P}_{\mathbf{2V}}{\varvec{\Sigma }}^{-\mathbf{1}}. \end{aligned}$$

Here, $\mathbf {U^*} \in \mathbb {C}^{(N-1) \times mn}$ and $\mathbf {V} \in \mathbb {C}^{(N-1) \times (N-1)}$ are the conjugate transpose of $\mathbf {U}$ and $\mathbf {V^*}$, respectively; and $\varvec{\Sigma }^{-\mathbf{1}} \in \mathbb {C}^{(N-1) \times (N-1)}$ denotes the inverse of the singular values $\varvec{\Sigma }$. By replacing $\varvec{\Psi } = \mathbf {U} \omega$ in Eq. (6), i.e. $\mathbf {A(}\varvec{\Psi )} \approx \varvec{(\Psi )}\sigma$, we obtain the dynamic modes $\varvec{\Psi }$. $\because \mathbf {U} = \mathbf {P}_{\mathbf{2V}}{\varvec{\Sigma }}^{-\mathbf{1}}$; therefore, we have:

9 $$\begin{aligned} \varvec{\Psi } = \mathbf {P}_{\mathbf{2V}}{\varvec{\Sigma }}^{-\mathbf{1}}\omega \end{aligned}$$

The complex eigenvalues $\sigma$ contain growth/decay rates and frequencies of the corresponding DMD modes [26, 27]. If $\sigma _j$ are the diagonal elements of $\sigma$ from Eq. (5), the temporal behaviour of the DMD modes is then formed via Vandermonde matrix $\mathcal {V}$, which raises its column vector to the appropriate power. $\mathcal {V}(f)$ with $(N-1)\times (f+1)$ elements will be defined as follows:

10 $$\begin{aligned} \mathcal {V}(f) = \begin{pmatrix} 1 &{} \sigma _1^1 &{} \sigma _1^2 &{} ... &{} \sigma _1^f \\ 1 &{} \sigma _2^1 &{} \sigma _2^2 &{} ... &{} \sigma _2^f \\ \vdots &{} \vdots &{} \vdots &{} \vdots &{} \vdots \\ 1 &{} \sigma _{N-1}^1 &{} \sigma _{N-1}^2 &{} ... &{} \sigma _{N-1}^f \\ \end{pmatrix}, \end{aligned}$$

$\mathcal {V}(N)$ is a standard Vandermonde matrix for reconstruction but if $f>N$, this is used for forecasting. DMD modes with frequencies $\mu _{j}$ is defined by:

11 $$\begin{aligned} \mu _{j} = \frac{\ln (\sigma _{j})}{\delta {t}}, \end{aligned}$$

where $\delta {t}$ is the lag between the images. The real part of $\mu _{j}$ regulates the growth or decay of the DMD modes, while the imaginary part of $\mu _{j}$ drives oscillations in the DMD modes.

### Ordering dynamic modes

In order to select the most significant dynamic modes, the method suggested in [7, 12] is to calculate the logarithmic values of the $diag(\sigma )$. The frequencies which are near origin are the most significant modes. The other way we propose here is by calculating the phase-angles for the complex eigenvalues.

The absolute value for the phase-angles are calculated and modes with unique phase-angles are selected. Doing this will remove one of the conjugate pairs in the dynamic modes. These conjugate modes have same phase-angles but with different signs and look and capture similar information [24]. After discarding one of the conjugate pairs, the dynamic modes are then sorted in ascending order of their phase-angles. The resultant dynamic modes are thus sorted according to their significance. In this study, we have considered the first three significant dynamic modes when reconstructing the original sequence.

### Reconstruction from DMD modes (R-DMD)

The novel reconstruction DMD aims at reconstructing the image sequence from the dynamic modes. This can be achieved in a least squares solution.

12 $$\begin{aligned} \left\| \varvec{\Psi } - \hat{\mathbf{P}_\mathbf{2}}\mathbf{V}\varvec{\Sigma }^{-\mathbf{1}}\omega \right\| \end{aligned}$$

Therefore, the reconstruction of the original image sequence can be formulated as follows:

13 $$\begin{aligned} \hat{\mathbf{P}_{\mathbf{2}}} = {\varvec{\Psi }}\omega ^{-\mathbf{1}}{\varvec{\Sigma }} \mathbf{V}^{-\mathbf{1}} \end{aligned}$$

Since the contrast changes are captured in the most significant modes and motion components in the least significant ones, it is desirable to discard the least significant modes. The crux of making this work is to select the *K* modes that are contributing to the contrast changes, and not the motion changes.

The original sequence is thus constructed using first *k* significant modes along with their corresponding eigenvectors from $\widetilde{H}$. The algorithmic details of our approach is described in Algorithm 1.

14 $$\begin{aligned} \hat{\mathbf{P}_\mathbf{2}} = {\varvec{\Psi }}_{\{\mathbf{1..k}\}}\omega ^{-1}_{\{\mathbf{1..k}\}}{\varvec{\Sigma }} \mathbf{V}^{-\mathbf{1}} \end{aligned}$$

### Windowed-DMD (W-DMD)

The Windowed version of DMD method runs DMD over a window of consecutive images in a sequence in an overlapping fashion. The output of DMD at each window produces $W-1$ dynamic modes where *W* is the length of the window. To compensate the periodic free breathing from the DCE-MRI sequence in this study, we consider *W* = 3. For instance, running DMD on the window containing the first three images in the sequence, we obtain two dynamic modes (in general for *N* images, we get $N-1$ DMD modes [33]). The first dynamic mode ‘c1’ captures the low-rank image across the window and the second dynamic mode ‘c2’ captures the sparse representation, which essentially contain motion artefacts pertaining to periodic free breathing. In the next step, we exclude the first image and consider images $\{2, 3, 4\}$, followed by $\{3, 4, 5\}$ and $\{4, 5, 6\}$ and so on as shown in Fig. 3. Finally, we concatenate all of the c1s across all the windows to obtain W-DMD component-1 (W-DMD (C1)), and similarly, concatenation of the c2s produce W-DMD component-2 (W-DMD (C2)) [31]).
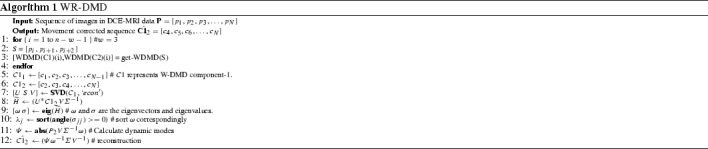


### Comparisons with DMD and MR-DMD

Dynamic mode decomposition (DMD), in computer vision, has been introduced for robustly separating video frames into a background model and foreground components [7]. The DMD method has been applied on a data matrix containing image sequence from a surveillance video. The DMD eigenvalue frequencies near the origin are interpreted as background (slow varying modes) portions of the given image sequence, and the frequencies bounded away from the origin are their sparse counterparts (fast varying modes). Specifically, the parts in image sequence that do not change in time have an associated frequency $\Vert \mu _{j}\Vert \approx 0$, which corresponds to background.

DMD with a multi-resolution [12] approach decomposes an image sequence into multi-time scale features and objects. The MR-DMD approach is similar to that of applying standard DMD technique at several resolutions on fast varying dynamic modes after discarding the background modes or the slow varying modes. Thereby allowing an image sequence to be separated into objects moving at different rates against the slowly varying background, thus allowing for multiple target tracking and detection. MR-DMD method has efficiently demonstrated, shifting $El\,\, Ni\tilde{n}o$ from ocean temperature data.

**Fig. 4:**
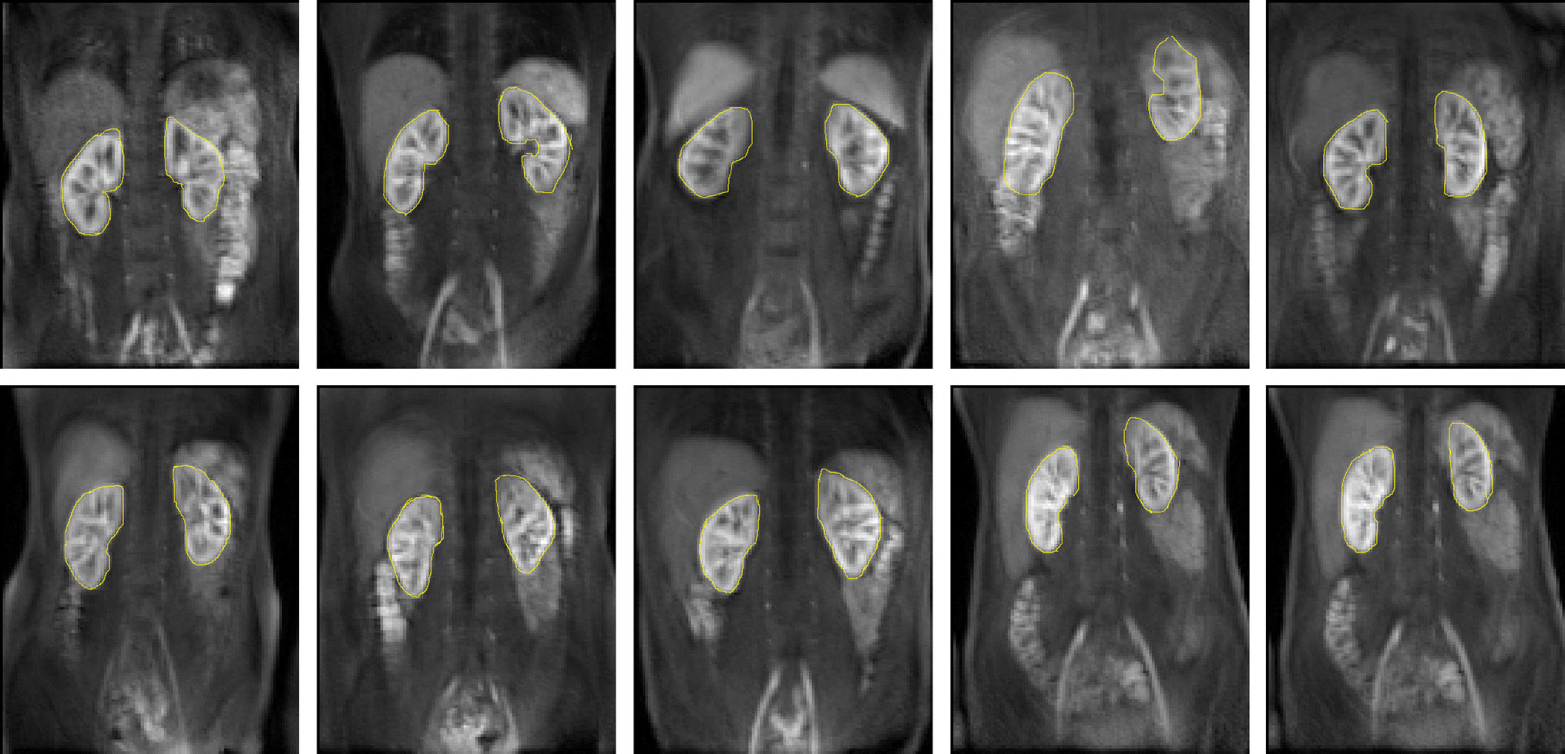
Exemplars of dynamic MR images from 10 healthy volunteers’ kidney slice produced by DCE-MRI sequence considered as 10 different data sets in this study. The images here show the central kidney slice at time 120*s* aortic peak enhancement after the contrast agent is injected. The *yellow* boundary on the kidneys is a result of manual delineation from a human expert. The mean intensity values are calculated in this region across the time producing time–intensity plots (colour figure online)

The MR-DMD framework implicitly has a windowing architecture since it implements the standard DMD on the windows of fast varying modes iteratively. In contrast, our proposed method is essentially different. Our proposed W-DMD method runs standard DMD over an overlapping window of consecutive images producing low-rank and sparse modes at each window of the image sequence. The consecutive set of low-rank images forms the W-DMD component-1 (C1), whereas W-DMD component-2 (C2) reveals the sparse components. The pseudo-periodic free breathing motion from the DCE-MRI sequence is thus compensated by discarding the sparse components, i.e. W-DMD(C2).

Later, this W-DMD (C1) is given as an input to standard DMD algorithm to produce set of most significant modes (slow varying) capturing the contrast changes, and least significant modes (fast varying modes) capturing motion components. Using the first three significant DMD modes, the original sequence is then reconstructed via our R-DMD method. Thus, utilising the pipeline of W-DMD and R-DMD we introduce WR-DMD for the first time to carry out movement correction in medical image sequences in a manner that is both extremely efficient and completely data driven.

## Data set

The data sets obtained were from 10 healthy volunteers’ as shown in Fig. 4 acquired after injection of 0.05 *mmol* / *kg* of Gd-DTPA (Magnevist) contrast agent, on a 1.5*T* Siemens Avanto MRI scanner, using a 32-channel body-phased array coil. The MRI acquisition sequence consisted of a 3D spoilt gradient echo sequence utilising an echo time, TE of 0.6ms, repetition time, TR of 1.6ms, and a flip angle, FA = 17 degrees with a temporal resolution of 1.5*s* collected for 180*s*. The acquired DCE-MRI data sets cover the abdominal region, enclosing left and right kidneys and abdominal aorta.

## Experiments and results

In this section, we discuss our experimental procedure along with the results. The objectives of our experiments are as follows:

1. *Selection of optimal window length (W)* The W-DMD algorithm needs parameter *W*, i.e. the window length to run standard DMD. The objective of this experiment is to determine the optimal *W* which depends on the breathing cycle of the volunteer.
2. *Importance of W-DMD method* To show the importance of W-DMD which constitutes a simplified variant of the proposal in our two-step approach, we evaluate our results with and without using the W-DMD method. Since, the W-DMD method can compensate for the pseudo-periodic free breathing from the contrast-enhanced images by discarding sparse components, we hypothesise that without using it, the reconstruction results may contain some flickering effects. Therefore, we wish to examine whether using windowed-DMD on a sliding sequence of 3 consecutive images will really stabilise these breathing motion artefacts.
3. *Qualitative assessment of dynamic information captured by DMD* From the outset, we hypothesise that DMD can capture both large scale dynamics related to contrast changes and small-scale dynamics related to motion artefacts and noise. Therefore, we wish to examine whether these features are indeed picked up by DMD or not.
4. *Reconstruction using most significant modes* Since DMD can capture the contrast-enhanced images in the most significant dynamic modes and motion in the least significant, we hypothesise that reconstruction with the top three most significant DMD modes could provide a perfectly aligned image sequence.
5. *Effect of mode selection in the reconstruction* It is of interest to investigate the impact of the most significant dynamic modes on the generalisation performance of the stability within the image sequence. For this reason, we consider modes in $M\times 4$ as a set where *M* ranges from $1,\ldots ,16$. We hypothesise that stability within the image sequence should decrease with an increasing number of modes selected for reconstruction.
6. *Comparison with other registration methods* We wish to examine the performance strength of our approach by comparing with two registration methods from the literature.

### Evaluation

The evaluation of our experiments is based on two perspectives.

1. From the clinical perspective, we would like to examine the feasibility of using our approach as a means for removing respiratory motion artefacts from the dynamic image sequence containing dramatic regional changes in intensity due to contrast agent flow affecting the quality of the resulting time–intensity curves used for analysis. The curves are produced by calculating the mean intensity of the target ROI in an image, which in this case is based on the kidney. ROI analysis using time–intensity curves containing respiratory motion artefacts may affect subsequent compartmental model fitting, as the motion may also obscure subtle time–intensity features. Therefore, we have considered the smoothness of the time–intensity curve as a surrogate metric for quality of motion compensation. The smoother this curve is, the better the performance.
2. From the signal processing and computer vision perspective, we would like to examine the strength of our approach by calculating the mean motion magnitude across the DCE-MRI sequence. Since, the respiratory motion represents an obfuscating issue within the dynamic image sequence, it distracts attention away from, and potentially masks, areas that may exhibit subtle pathology within the image. For this purpose, we evaluate the motion between two consecutive images over the entirely reconstructed dynamic sequence in order to characterise the amount of motion as an indication of the stability of the dynamic sequence. This criteria is evaluated using a method called block-matching-block motion estimation [1]. A well-reconstructed dynamic sequence should have a smaller overall global mean.

### Selection of optimal *W* (window length)

In order to determine optimal *W*, we would like to see whether there exists any periodicity in the motion. For this purpose, we calculate the motion amongst the images in the sequence with respect to the first image using block-matching algorithm. This algorithm estimates the motion between two images using ‘blocks’ of pixels, i.e. by matching the block of pixels in image *K* to a block of pixels in image $K'$ by moving the block of pixels over a search region. The block subdivides the image *K* in block sizes [height width] and Overlap [*r c*] parameters. For each subdivision or block in image $K'$, the algorithm establishes a search region based on the maximum displacement [*r c*] parameter. The block searches for the new block location using an exhaustive search method [14]. We have considered [height, width] = [15, 15] and [*r*,*c*] = [5, 5].

**Fig. 5:**
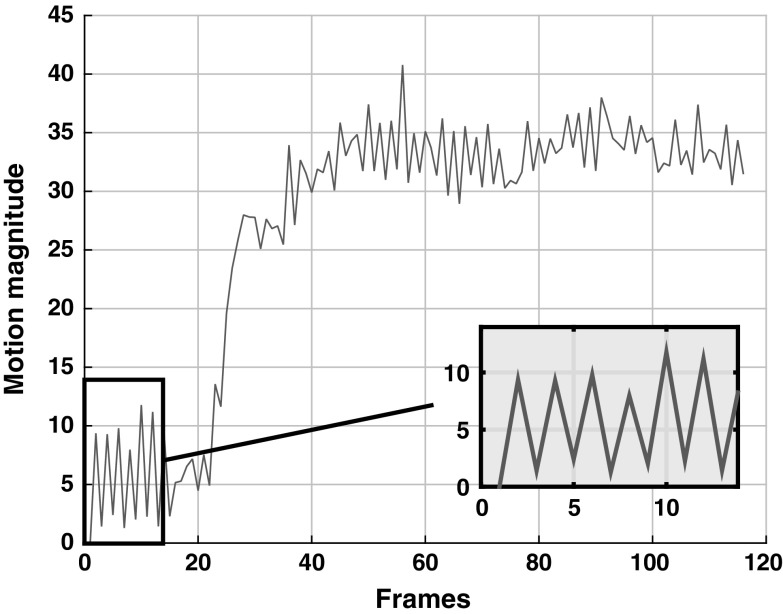
Motion magnitude with respect to first image in the sequence across the data set-1

The motion magnitudes across the data set-1 with respect to first image of the sequence is shown in Fig. 5. It can be clearly seen that the motion is periodic for every three consecutive images. Therefore, we have chosen a window length of 3 to conduct our experiments.

**Fig. 6:**
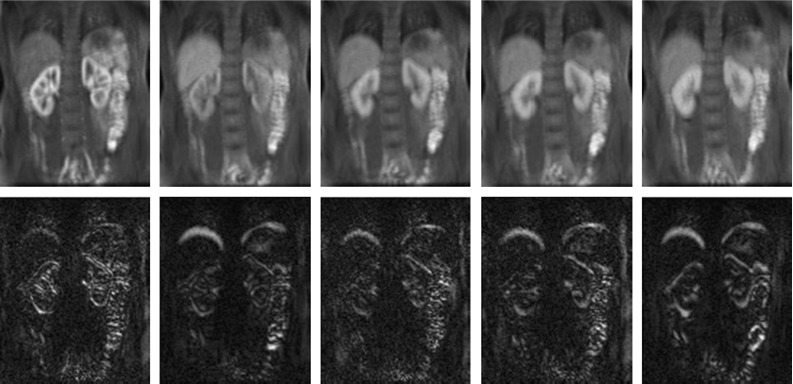
(*Top*) five images from data set-1’s W-DMD(C1) at time = {30,50,74,82,100}s showing the low-rank images. The *first image* shows the peak stage of contrast agent inside the kidney region. (*Bottom*) Corresponding images from the W-DMD(C2) showing their sparse representation

**Fig. 7:**
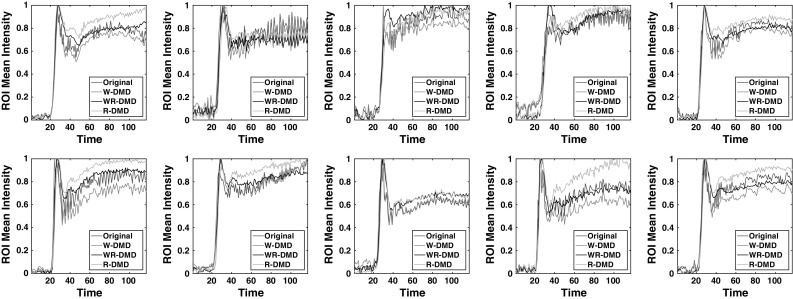
Time–intensity curves across 10 data sets (sequentially from *left* to *right*)

### Performance of W-DMD

Each of the 10 healthy volunteers’ DCE-MRI dynamic sequences consisting of 120 images are given as an input to W-DMD algorithm sequentially. The DMD algorithm at each sliding window consisting of three images produces two dynamic modes namely W-DMD component-1 revealing the low-rank image and W-DMD component-2 revealing the sparse image as shown in Fig. 6. All the low-rank images are then concatenated to form a sequence of 118 images called W-DMD component-1 (W-DMD(C1)) and similarly the concatenation of sparse images form W-DMD(C2).

For the purpose of clinically relevant evaluation, an expert (radiologist) manually delineated the kidney ROIs across the 10 data sets as shown in Fig. 4. Using these ROIs, the time–intensity curves are obtained. The curves for W-DMD(C1) for the right kidney shows a little reduction in intensity variations as seen in Fig. 7 (W-DMD (red)). The evaluation results after processing with block-matching algorithm depicts a decrease of $80.54\%$ average motion magnitude against the original sequences across the 10 data sets as shown in Fig. 9.

**Fig. 8:**
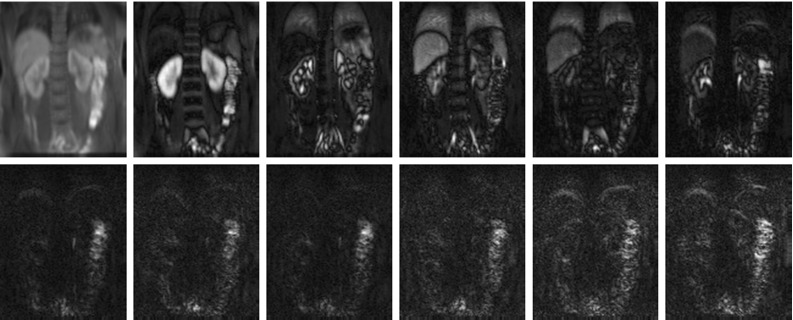
(*Top*) The top six most significant DMD modes on W-DMD(C1) from data set-1. (*Bottom*) bottom six least significant DMD modes on W-DMD(C1)

### Importance of W-DMD

**Fig. 9:**
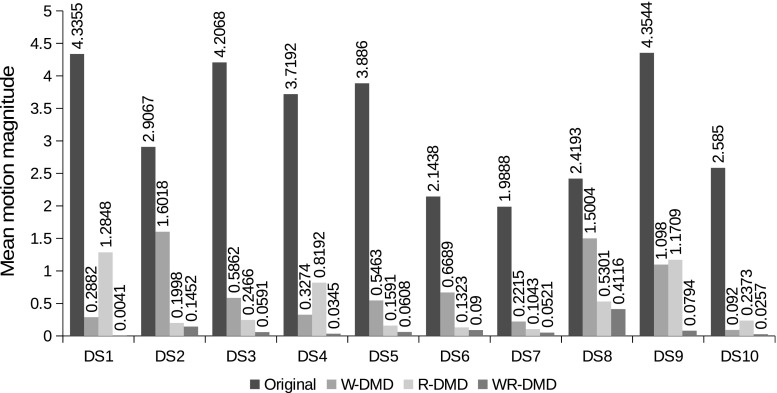
A comparison of mean motion magnitude between original and W-DMD-, R-DMD- and WR-DMD-processed image sequences. The results are shown across 10 data sets calculated using block-matching-block algorithm. A smaller mean motion magnitude indicates a more stable sequence

In order to examine the strength of W-DMD, we directly input each of the ten healthy volunteers’ DCE-MRI dynamic sequences to the standard DMD algorithm. Using the top three most significant modes, the original sequence is reconstructed using our R-DMD method. The results in Fig. 9 show greater motion magnitude when compared to results obtained through W-DMD on the data sets $\{1,4,9,10\}$. The time–intensity plots in Fig. 7 from data sets $\{2,3,5,6,7,9\}$ reveal a greater amount of fluctuations from the graphs produced by the R-DMD method even though the results of their mean motion magnitude are lower when compared to W-DMD method. This proves our hypothesis that although excluding the W-DMD step stabilises the motion globally, the periodic free breathing would still remain locally. Therefore, discarding the sparse components, i.e. W-DMD(C2) eliminates periodic free breathing from the contrast-enhanced images.

### Qualitative assessment of DMD

In the next step, the W-DMD(C1) containing the low-rank images are then given as an input to DMD algorithm producing 117 dynamic modes. The first mode reveals the low-rank model across all the images and the remaining 116 modes capture the sparse representations. The contrast changes are captured in the most significant modes, in particular, mode-2 capturing kidney region and mode-3 and 4, spleen and the liver regions, respectively, as shown in Fig. 8 (top) for data set-1.

Noise and residuals including the motion components are captured in the least significant modes [Fig. 8 (bottom)].

### Reconstruction using most significant modes

The first three modes are selected for the reconstruction of the W-DMD(C1) sequence of images, discarding the rest of the 113 modes. The reconstructed W-DMD(C1) using the WR-DMD algorithm produces a very promising and stable image sequence compensating for all the complex movements. The qualitative results obtained from the WR-DMD (black) in Fig. 7 show smoother curves in the time–intensity plots when compared to the original sequence and W-DMD(C1) sequence. Complete complex movement artefacts arising due to respiration, pulsation and involuntary movements are all compensated through the WR-DMD reconstructed images as shown in Fig. 9. A decrease of $99\%$ average motion magnitude can be seen against the original sequences across the 10 data sets.

### Effect of mode selection in the reconstruction

**Fig. 10:**
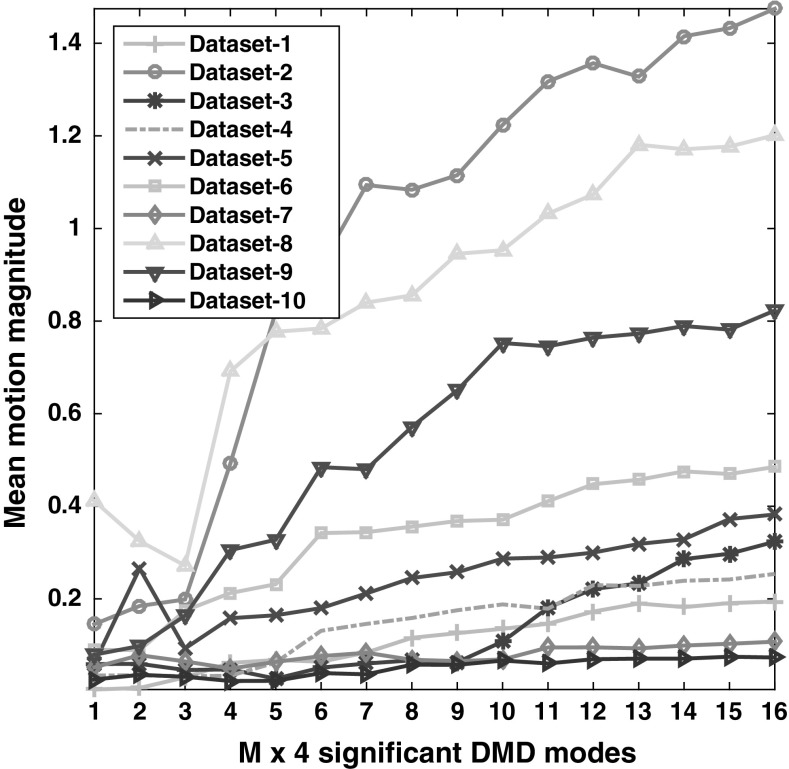
Mean motion magnitude across 10 data sets calculated using block-matching-block algorithm utilising WR-DMD. A *smaller* mean motion magnitude indicates a more stable sequence. Here, M is the number of modes that were used in the reconstruction process. As the number of modes increases, the mean motion magnitude also increases

Since reconstruction needs to operate with significant modes, it is of interest to find out the minimum number of modes required. We hypothesise that a greater number of modes should result in less stable performance; however, at the same time, we would like to know the minimum number of modes that are required to reconstruct the original sequence. Consequently, we select the windows of the first $\{4,8, \ldots 64 \}$ images. We can consider at most 64 modes because one member of each conjugate pairs of the DMD mode is redundant. Consequently, we are left with 61–62 modes. The results in Fig. 10 reveal that as the number of modes increases, the mean motion magnitude also increases, consistent with our hypothesis that a greater number of modes results in increased motion variance (methodologically, we would expect the optimal number of DMD modes to be data dependent).

### Comparison with other registration methods

In order to compare our approach with registration-based methods, we should follow a gold standard approach that is manually delineating the target region and performing the rigid registration, which requires a human expert and is inconvenient. Nevertheless, to obtain a fair comparison, a human expert manually delineated the area around the right kidney region. Therefore, we have selected only this part of the delineated region for the registration and not the whole image. We have opted to use intensity-based methods such as Affine and translation registration methods for our comparison, since these methods are less sensitive to contrast change, and their implementation is freely available.

Evaluating the performance of our approach on DCE-MRI data is difficult due to the lack of the prior knowledge on contrast change and the motion.

One way of evaluating the performance is to calculate the total variation score given by the estimate of the standard deviation of the consecutive *m* time–intensity data points $\hat{\mathbf{t}}_{[i]}~\forall i \in \{1, \ldots , m\}$. Smaller values indicate smoother time series. However, this is scale variant and depends upon the application. Therefore, to induce scale invariance, we evaluate the ‘degree of smoothness’ score of the total variation score calculated from the time–intensity data points by dividing it by the absolute mean of the difference of the consecutive time intensity data points. The difference is used to avoid the assumption of stationarity in the data and it is calculated as $\widetilde{\mathbf{t}}_{[i]} \leftarrow \hat{\mathbf{t}}_{[i+1]} - \hat{\mathbf{t}}_{[i]},~\forall i \in \{1, \ldots , m\}$.

The degree of smoothness score $\mathbf {D}$ is thus given by:

15 $$\begin{aligned} \mathbf {D} = \frac{std(\widetilde{\mathbf{t}})}{abs(mean(\widetilde{\mathbf{t}}))}. \end{aligned}$$

**Fig. 11:**
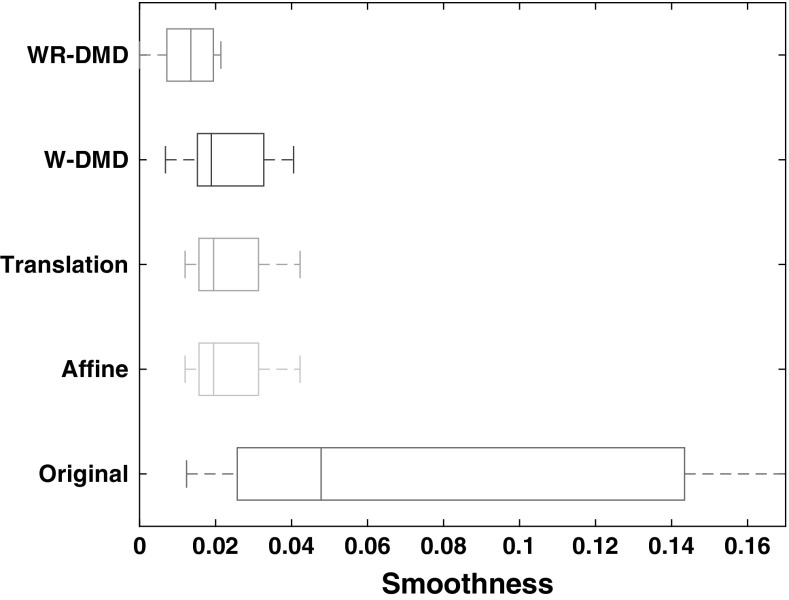
Comparison with intensity-based image registration methods. Each *box-plot* has 10 values corresponding to 10 data sets. A smaller value in the X-axis indicates greater smoothness; so smaller is better

The results in Fig. 11 clearly show WR-DMD outperforms the standard registration methods.

## Conclusions, discussions and future directions

This study shows the significance of WR-DMD approach, as a viable movement correction algorithm for processing DCE-MRI data. We applied the WR-DMD approach on 10 data sets of DCE-MRI data collected from healthy volunteers. The proposed algorithm has very low time complexity^1^. In addition, compared to existing methods, e.g. RDDR & STICA, it has the advantage of requiring no parameter tuning. W-DMD can extract low-rank and sparse representations within an image sequence. The motion artefacts for periodic free breathing are captured in the sparse components. The low-rank W-DMD component-1 is then given as an input to the standard DMD algorithm producing dynamic modes. We found that the contrast changes are captured in the most significant dynamic modes and motion in the least significant ones. The original sequence is then reconstructed utilising the top three most significant dynamic modes using R-DMD. The results demonstrate that the proposed WR-DMD method is a promising approach for correcting respiratory or similar motions in complex dynamic medical image sequences containing significant temporal intensity changes due to contrast agent uptake or other comparable mechanisms.

The major point of discussion would be in answering whether the WR-DMD framework be sufficiently robust when it comes to scanning patients who tend to be more susceptible to motion artefacts? From the clinical point of view, this can only be answered with a conjecture as we have no patient data but we would argue ‘yes’ [29]. This question provides a good opportunity to recognise that both volunteers and patients will be susceptible to motion. From computer vision and signal processing point of view, W-DMD, in this study has been demonstrated for removing pseudo-periodic breathing motion. It is always necessary to check whether there exists any pseudo-periodic breathing in the data set. For this purpose, motion amongst the images in the sequence with respect to the first image should be calculated using block-matching algorithm as discussed in Sect. 4.2. Later, the number of images for which the motion is periodic should be determined. For example, in Sect. 4.2 we show that the motion was periodic for every three images in our data sets, and hence, we set W = 3 as a parameter in W-DMD to conduct our experiments. Similarly if the periodicity in the motion is observed for ‘*n*’ images for a patient/ healthy volunteer, especially children, who tend to be more susceptible to motion artefacts, we conjecture that making the window length adaptive to ‘*n*’ might be sufficient to tackle the pseudo-periodic motion.

The adaptive version of the W-DMD forms our future work. In addition to that, DMD, in this study, also has demonstrated to extract dominating regions of causally-connected intensity fluctuations. In this context, perfusion inside the kidney region is the most dominating region with the intensity fluctuations due to the injection of contrast agent. DMD was thus able to naturally capture the kidney region as mode-2 followed by liver and spleen in the other modes. Therefore, in our future work we would like to perform segmentation of the kidney region of interest for automatically quantifying the kidney function. Finally, we would like to explore the possibility of using our proposed methodology in automatically correcting for the movements in ‘coloured’ [32] aerial images which are captured of the same terrain but on different days.

## Electronic supplementary material

Below is the link to the electronic supplementary material.

Supplementary material 1 (avi 626 KB)

Supplementary material 2 (avi 629 KB)

Supplementary material 3 (avi 648 KB)

Supplementary material 4 (avi 4740 KB)

## Footnotes

1 That is, image sequence containing 120 frames each of size $120\times 104$ takes about 3–5 s to compute on a desktop computer with 8GB of RAM memory running Intel(R) Core(TM) i5-4590 CPU @ 3.30GHz.
